# Dietary Polyphenols: Extraction, Identification, Bioavailability, and Role for Prevention and Treatment of Colorectal and Prostate Cancers

**DOI:** 10.3390/molecules27092831

**Published:** 2022-04-29

**Authors:** Naveed Ahmad, Muhammad Qamar, Ye Yuan, Yasir Nazir, Polrat Wilairatana, Mohammad S. Mubarak

**Affiliations:** 1 Multan College of Food & Nutrition Sciences, Multan Medical and Dental College, Multan 60000, Pakistan; 2 Institute of Food Science and Nutrition, Bahauddin Zakariya University, Multan 60800, Pakistan; muhammad.qamar44@gmail.com; 3 Institute for Molecular Bioscience, The University of Queensland, Brisbane, QLD 4072, Australia; ye.yuan1@uq.net.au; 4 Department of Chemistry, Faculty of Sciences, University of Sialkot, Sialkot 51300, Pakistan; ynchem@yahoo.com; 5 Department of Clinical Tropical Medicine, Faculty of Tropical Medicine, Mahidol University, Bangkok 10400, Thailand; 6 Department of Chemistry, The University of Jordan, Amman 11942, Jordan

**Keywords:** polyphenols, extraction technique, bioavailability, apoptosis, colorectal cancer, prostate cancer, cell viability, minimum half-inhibitory concentration

## Abstract

Fruits, vegetables, and other edible plants in our diet have numerous health benefits, due to the bioactive compounds in these food items, including polyphenols. These plants are a rich and promising source of natural products and phytochemicals that can be used to treat and prevent numerous diseases and prevent the progression of cancer. Dietary polyphenols exhibit chemo-preventive and therapeutic effects against various ailments, including several types of cancer. The current study focuses on polyphenol’s traditional and advanced extraction methods, with supercritical extraction as a novel approach. It also deals with their identification, bioavailability, and role in preventing and treating colorectal and prostate cancers. Additionally, the article covers the literature that deals with the anticancer activities of polyphenols, as well as their potential use as anticancer agents.

## 1. Introduction

Cancer continues to be a global burden and leading cause of mortality, despite many technological and pharmaceutical advances over the past two decades [1]. According to estimates from the World Health Organization (WHO), in 2019, cancer ranked as the first or second leading cause of mortality before the age of 70 years in 112 of 183 countries and was third or fourth in a further 23 countries [2]. In addition, there were 19.3 million new cancer cases diagnosed, and the death toll reached 10.0 million in 2020. Colorectal (10.0%) and prostate (7.3%) were the most diagnosed cancers, after breast (11.7%) and lung (11.4%) cancers. According to these estimates, it is predicted that lung cancer was responsible for 1.8 million deaths (18 %), making it the leading cause of mortality. Moreover, it is predicted that the number of new cancer cases will rise to 28.4 million by 2040, posing a severe threat to human health [2]. Cancer treatment methods include surgery, radiotherapy, chemotherapy (use of anticancer drugs), and other specialized techniques. According to published reports, approximately 90–95% of all cancers are attributed to lifestyles, such as alcohol consumption, obesity, outdoor pollution, and food additives; the remaining 5–10% are due to defective genes [3]. 

Prostate cancer occurs in a man’s prostate, a small walnut-shaped gland that produces the seminal fluid that nourishes and transports sperm. It is the second most common cancer diagnosed globally, after lung cancer, and it is more common in men older than 50. Age and family history are the key contributing factors to this risk factors for prostate cancer [4]. On the other hand, colorectal cancer refers to the slowly developing cancer beginning as a growth, called a polyp, inside the colon or rectum. Colorectal cancer signs and symptoms may include blood in the stool, changes in bowel movements, weight loss, and fatigue [5]. Colorectal cancer is one of the most common and preventable cancers globally. The primary risk factors involve smoking, excessive alcohol consumption, obesity, and others [6]; the risk of developing colorectal cancer rises after age 50. Colorectal cancer ranks as the second most lethal cancer and third most prevalent malignant tumor worldwide. In 2018, 1.8 million new cases were detected and 881,000 deaths were reported; new cases may increase to nearly 2.5 million in 2035 [6]. Treatment involves surgery, radiotherapy, and chemotherapy. Current chemotherapy includes both single-agent therapy, mainly fluoropyrimidine (5-FU)-based and multiple-agent regimens, containing one or several drugs, including oxaliplatin (OX), irinotecan (IRI), and capecitabine (CAP, XELODA, or XEL) [7]. 

On the other hand, a healthy diet plays a crucial role in maintaining a healthy life. In this regard, the consumption of fruits and vegetables has numerous health benefits, due to bioactive compounds, including polyphenols, carotenoids, flavonoids, and phytosterols, in these food items [8,9]. Moreover, dependence on intrinsic foodstuffs is gaining popularity in the fight against diseases, such as cancer, cardiovascular disorders, diabetes, and others [10]. In addition, plant-derived chemicals have attracted the attention of scientists, dietitians, and medicinal chemists, due to their numerous health-promoting effects. These naturally-derived compounds can be used in the fight against diseases, such as cardiovascular disorders and cancer insurgence, among others, and have attracted scientific and medical communities due to their lesser side effects and cost in comparison to chemotherapy [11]. Naturally occurring polyphenols have been particularly used in traditional medicine to treat numerous illnesses [12]. These compounds play significant roles in reducing cancer; they can be used as therapeutic agents in treating and preventing diabetes, obesity, cardiovascular diseases, oxidative stress, asthma, and microbial infections. In this context, we have published comprehensive reviews with many references about some polyphenols found in fruit and vegetables, including resveratrol [13,14], kaempferol [15], and quercetin [16]. Polyphenols are the most widely researched class of functional biomolecules, benefiting in gut health, hypertension, cancer prevention, incendiary infections, metabolic and neurodegenerative illnesses, and diabetes, along with antioxidative potential [12].

Primary cancer treatment can be classified into chemotherapy, radiotherapy, and surgery. In chemotherapy, the toxicity and non-targeted features of some drugs cause damage to both cancer and healthy cells also leading to severe side effects. Therefore, alternative treatment is necessary [17]. As a result, cancer treatment and prevention have been hot topics for researchers. Natural polyphenols have gained attention for their anticancer effects in the last two decades and have been a potential candidate for anticancer drugs. Polyphenols are generated by the metabolism of plants [18] and are present in significant amounts in fruits, vegetables, spices, soy, nuts, and beverages of plant origins. These can be divided into five classes, based on their structure, including flavonoids, phenolic acids, lignans, stilbenes, and other polyphenols. Flavonoids and phenolic acids are the primary polyphenols in plants and account for 30% and 60% of all natural polyphenols, respectively [19]. As polyphenols exhibit anti-inflammatory, antioxidant, and, especially, antineoplastic activities, they have the potential to serve as a cost-effective, non-toxic, and non-invasive replacement for chemotherapy [20,21].

Much research has been done to express the effects of polyphenols against cancers. Through a Canadian case-control study, Christensen et al. reported the possible increased risk of lung cancer by a low intake of flavonoids from food sources [22]. Similarly, Woo et al. performed a case-control study in Korea, and the result showed that dietary flavonoids could decrease the risk of gastric cancer, which is more prominent in women [23]. Other investigations indicated that the risk of colorectal cancer was reduced by consuming dietary polyphenols [24,25,26]. Moreover, Ghanavati et al. reported that the consumption of polyphenols is inversely associated with prostate cancer [27].

The aforementioned epidemiological studies present the general relationship between polyphenol-rich foods and the risk of cancer, proving the potential anticancer activity of polyphenols. Based on the preceding discussion, this review focuses on the current knowledge of dietary polyphenols: extraction, identification, bioavailability, and their chemo-preventive and healing ability in treating and preventing colorectal and prostate cancers, along with their mechanisms of action. For this purpose, we obtained recent relevant references pertaining to these aspects of polyphenols from different databases, such as MEDLINE (PubMed), Google Scholar, ScienceDirect, Scopus, and SciFinder. We hope this review will be a valuable addition to the field and great help for researchers.

## 2. Extraction Techniques of Polyphenols

Extraction techniques are classified as the traditional and modern. The conventional methods include maceration, percolation, or Soxhlet extraction, which are still widely applied in phytochemical analysis. However, there are some disadvantages. The traditional way is usually time- and energy-consuming and requires a large amount of potentially toxic solvents and starting materials, due to the low polyphenols yield, leading to a considerable amount of waste. Besides, the traditional method is not suitable for thermolabile compounds, due to the extraction conditions. On the other hand, the modern extraction method includes ultrasound-assisted extraction (UAE), microwave-assisted extraction (MAE), and supercritical fluid extraction (SFE). These techniques can significantly improve the extract efficiency to achieve a high polyphenols yield, thus reducing extraction time and solvent consumption [28]. Table 1 compares the traditional and modern extraction methods, in terms of time, solvent use, cost, sample size, efficiency, and yield. 

Ultrasound-assisted extraction (UAE) is a simple and efficient method to extract polyphenols with brief extraction time and low solvent usage. It can release the bioactive components through the disruption of cells by the acoustic cavitation effect [29]. However, this method has a high requirement of the sample particle size to achieve better extraction efficiency [30]. UAE is widely applied in extracting phenolic and flavonoid antioxidant compounds to recover a high yield [31]. Additionally, this method is suitable for thermolabile compounds, as the procedure is usually performed under room temperature conditions [32]. Saifullah et al. reported the extraction of lemon-scented tea tree (*Leptospermum petersonii*) leaves, through shaking water bath extraction and UAE, and found that the UAE method (using 50% acetone in water) is more efficient in extracting phenolics (98.91 mg GAE/g DW), compared to shaking water bath extraction (85.81 mg GAE/g DW). At the same time, these two methods achieved similar yields for flavonoids and pro-anthocyanidins [33]. The same group made another comparison through the extraction of propolis, and the efficiency was measured through four aspects: extraction yield, total phenolic content, flavones and flavonol content, and flavanone and dihydroflavonol content. Results showed that double UAE (15 min at 20 kHz, 70% ethanol) achieved a higher yield (37.1–96.7 g balsam/100 g crude propolis), compared to double maceration (condition, 24 h at room temperature, 250 rpm; yield, 35.6–91.2 g balsam/100 g crude propolis) and double microwave treatments (condition, 1 min at 140 W; yield, 36.1–95.9 g balsam/100 g crude propolis) [34]. 

MAE is used to extract aqueous samples by fast heating in an oxygen-rich environment and reducing time and solvent usage [35]. Compared to classical heating, MAE can heat all samples simultaneously, gaining a high efficiency and extraction rate at a low cost. The effect is achieved by transferring microwave energy to the whole system through the solvent, in the form of heat [36]. Therefore, MAE has been a feasible option for natural polyphenols extraction. Sen and coworkers compared the polyphenols extraction yield of *Centella* leaves by using MAE and Soxhlet. The results indicated that MAE is better for obtaining total phenolic content, flavonoid content, and triterpenoids. It is energy-efficient and time-saving, as the extraction time was only 6 min, compared to 36 for Soxhlet, and the carbon load was 200 times less [37]. Moreover, Mohapatra and colleagues reported the effect of the extraction method and solvent system on the yield of the bioactive compounds from *Centella* leaves. The results showed that, by using methanol as the extraction solvent, MAE gained the highest recovery of eight bioactive compounds viz chlorogenic acid (0.18%), rutin (0.11%), quercetin (0.02%), kaempferol (0.10%), madecassoside (0.76%), asiaticoside (2.66%), madecassic acid (0.32%), and asiatic acid (1.98%) [38]. In terms of propolis extraction, Trusheva et al. found that MAE (800 W), with ethanol (75% *v*/*v*) as the extraction solvent, exhibited a better yield (73–75%) of flavanones and dihydroflavonols, as compared to UAE (41–53%) and maceration (55–58%). Besides, MAE was found to be the fastest extraction method, with only 2 × 10 s. compared to UAE (30 min) and maceration (72 h) [39]. 

SFE is the separation method using supercritical fluid, performed at atmospheric temperatures, which can help prevent thermal denaturation. The commonly used solvent is carbon dioxide, which is easy to remove [40]. Moreover, carbon dioxide is non-corrosive, colorless, and odorless, suitable for the food industry [41]. However, SFE has a high requirement for the phase equilibrium, pressure, and temperature to achieve a better yield and selectivity, which must be considered when selecting the method [42]. Radojkovic et al. found that SFE (carbon dioxide as the supercritical fluid) was more efficient in extracting non-polar compounds from *Morus alba* and *Morus nigra* leaves than Soxhlet with non-polar solvent (*n*-hexane), as the yield was 2.96 g/100 g for *M. alba* and 3.46 g/100 g for *M. nigra*, which was higher than that of Soxhlet (2.60 g/100 g for *M. alba* and 3.00 g/100 g for *M. nigra*) [43]. Another study showed that SFE using CO_2_ could achieve higher total tetrahydrocannabinol (THC) and total cannabidiol (CBD) recovery for the extraction of cannabis inflorescences, compared to ethanol maceration; however, the toxicity study showed that the SFE method could decrease half of the cytotoxic concentration, which needs to be considered when selecting an extraction method [44].

## 3. Bioavailability of Polyphenols 

**Figure 1:**
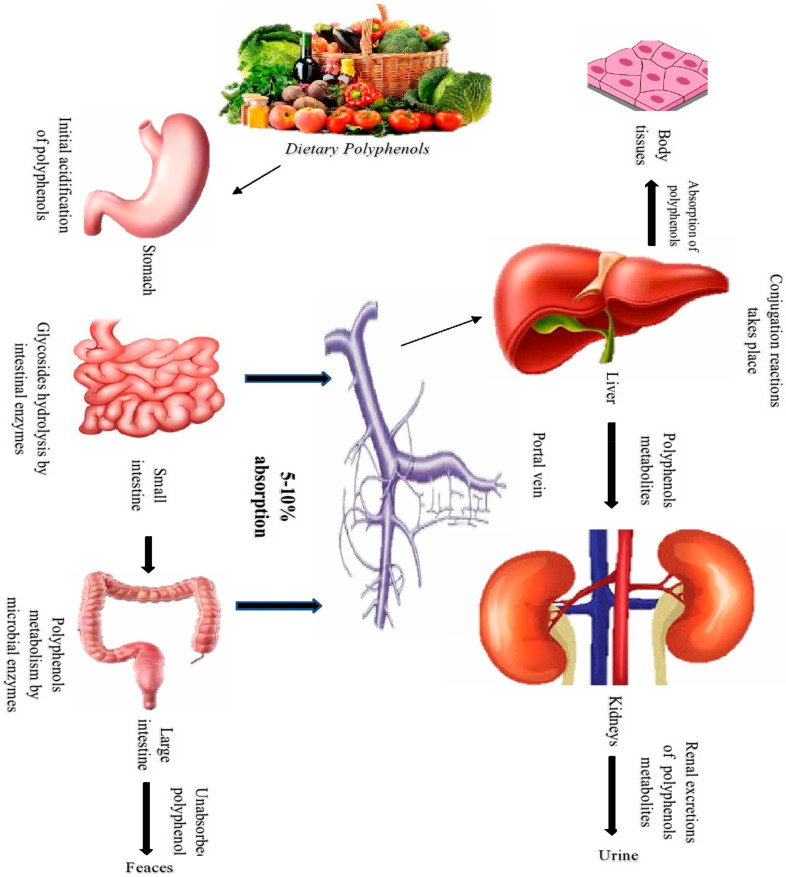
A sketch shows the bioavailability of polyphenols.

Foods containing polyphenols are used in a regular diet for good health. The metabolic processes, transport, and distribution of phenolics to their target organs might alter their structure and bioactivities. Biological access and bioavailability of polyphenols in the gastrointestinal tract (GI) are essential in their absorption (Figure 1). In addition, the polyphenols’ interaction with other dietary components affects their bio accessibility, whereas bioavailability refers to their capacity to be metabolized and dispersed throughout the body [45,46]. Polyphenols have low bioavailability, due to several characteristics that limit their metabolism, including solubility, chemical structural complexity, interaction with other compounds, and degree of polymerization. Along this line, the phytochemical’s bioavailability is described by McClements [47] as a variable that is influenced by four key factors:

BA = S* B* T* A*

where S* indicates the stability of the compounds after processing of food; B* indicates bioaccessibility (B*); T* is the number of molecules that remain intact after moving from the GI to the absorption site; and A* indicates the amount of actually absorbed compounds by the epithelium cells [48]. Glycosidase acts on glycosylated binding molecules in the oral cavity. In contrast, a significant release of polyphenols occurs in the stomach, and hydrolysis of some compounds may occur, due to the acidic medium (pH 2–4) [45,47]. After that, the GI performs most of the biotransformation of these substances, defining their bioavailability in the blood vessels before their absorption and circulation. Phases I and II of GI metabolization occurs in the gut and liver cells, respectively; microflora degradation can also occur in the colon. Phenolic molecules undergo oxidation, reduction, and hydrolysis in two phases, resulting in structural modifications, such as amino, carboxyl, and hydroxyl groups in phase I. On the other hand, phase II is involved in enzymatic processes, such as glucuronidation, sulfation, and methylation, to lower the toxicity of the chemicals and facilitate their removal [42,43,44,45,46,47].

After absorption, polyphenols in food are transported to the liver in amounts ranging from 5–10% [35]. Since most flavonoids are not decomposed by acid hydrolysis, but are degraded in an alkaline environment, pH shift from gastric (pH 2) to intestine (pH 6–7.5) affects the bio accessibility of flavonoids [46]. Accordingly, the amount of polyphenols consumed to exert their bioactivities in vivo is complicated. It extends beyond the link between the polyphenols consumed by the body and polyphenols excreted in the urine as these compounds are converted into other metabolites or degenerated along the way after several reactions [48]. Methods employed to overcome human metabolic obstacles and boost phenolic absorption involve food processing to increase bioavailability, the use of food products that protect phenols from degradation, and the improvement of delivery systems that protect compounds until they reach their destination, and they must carry out their bioactivities [45]. In this respect, the freezing technique improved the bio accessibility of anthocyanins (47.2% to 83.4%) and strawberry flavonoids (64.4% to 90.8%) [49]. Additionally, after a blanching procedure was given to highbush blueberry purée, the total quantity of anthocyanins in human blood increased [50]. Their stability improves when flavonoids are integrated into dietary matrices, including milk proteins. Similarly, green tea polyphenols in cheese were progressively released in the gastrointestinal environment from the tea-intra cheese, which slowed their breakdown and increased their antioxidant activity [51]. Furthermore, anthocyanins added to various food matrices such as milkshakes and omelets, shown similar effects of increased stability followed by oral phase, stomach, and intestinal digestions [52].

The use of delivery methods appears to be one of the most effective strategies for enabling polyphenols applications as therapeutic agents. This approach can improve the stability of these biomolecules under gastrointestinal processes and their ultimate absorption by epithelial cells [53]. Casein-, whey-, and zein protein nanoencapsulations are more effective than others in increasing the bio accessibility of flavan-3-ols and anthocyanin. Similarly, anticancer polyphenols such as resveratrol and quercetin, have enhanced bioavailability due to lipid-based nanoencapsulation. The real benefit of this delivery strategy is that it increases the solubility of hydrophobic substances in the GI tract. Numerous studies have shown that delivery systems such as liposomes, nanoemulsions, and polymeric/biopolymeric nanoparticles boost polyphenol efficacy in targeted cancer therapy [45].

## 4. Anticancer Activities of Dietary Polyphenols 

### 4.1. Prostate Cancer, Polyphenols, and Identification Techniques

Research findings showed that the polyphenol-rich sweet potato greens extract (SPGE) exhibits antiproliferative action against prostate cancer cell lines in vitro and in vivo subjects. In human prostate cancer PC-3 cells, SPGE flustered the cell cycle progression, reduced clonogenic survival, and modulated apoptotic regulatory molecules. The DNA degradation was appreciable by mediating the terminal deoxynucleotidyl transferase-dUTP-nick-end labeling (TUNEL) stain of the enhanced concentration of 3′-DNA ends. Yet, apoptosis appeared to be caspase-dependent, due to substrate cleavage. Similarly, the growth progression of prostate tumor xenografts was significantly inhibited by ∼69% in nude mice upon oral administration of 400 mg/kg SPGE, as shown by non-invasive real-time bioluminescent imaging and tumor volume measurement [54].

The effect of pomegranate whole juice and the ellagitannins-rich extract on gene expression for androgen-synthesizing enzymes and androgen receptor (AR) was measured. Genes expressions for steroid 5-α reductase type 1 (SRD5A1), 3-β-hydroxysteroid dehydrogenase type 2 (HSD3B2), and aldo-keto reductase family member C3 (AKR1C3) were measured against human prostate cancer cell lines (LNCaP, LNCaP–AR, and DU-145) for the respective androgen-synthesizing enzymes. The gene expression was notably suppressed two-fold. The most apparent gene expression inhibition and AR up-regulation by pomegranate polyphenols were observed in the LNCaP–AR cell line. Hence, gene expression inhibition for androgen-synthesizing enzymes, by pomegranate polyphenols, could be a promising niche to address androgen-independent prostate cancer and AR up-regulation in the human subclass [55]. Similarly, pomegranate peel polyphenols (PP) were tested for their in vivo apoptotic and antiproliferative effect on subcutaneous xenograft of human prostate cancer cells (PC-3) using a nude mouse model. An enzyme-linked immunosorbent evaluation was performed to detect the levels of vascular endothelial growth factor (VEGF) and cytokines tumor necrosis factor α (α-TNF). Reverse-phase, high-performance liquid chromatography with photo-diode array detection (RP-HPLC-PDA) was employed to quantify punicalagin, gallic, and ellagic acid inactive fractions. Results showed that PP significantly enhanced apoptosis and reduced tumor weight and volume in nude mice, with an increased α-TNF and decreased VEGF in the serum. Furthermore, RP-HPLC-PDA quantification revealed the respective concentrations of punicalagin, gallic, and ellagic acid at 407.0 ± 12.05, 8.917 ± 0.274, and 201.3 ± 3.544 mg/g [56].

A comparison between pomegranate juice and peel aqueous extracts was drawn to investigate their respective impact on prostate cancer, using the DU-145 and PC-3 cell lines. Peel extracts, compared to juice, exhibited more profound effects in inhibiting cell proliferation, colony formation, and migration. After treating the cancerous cells with pomegranate juice and isolated peel extracts, the growth-related mTOR/S6K signaling pathway was profoundly inhibited, confirming the anti-cancerous effects [57]. 

**Figure 2:**
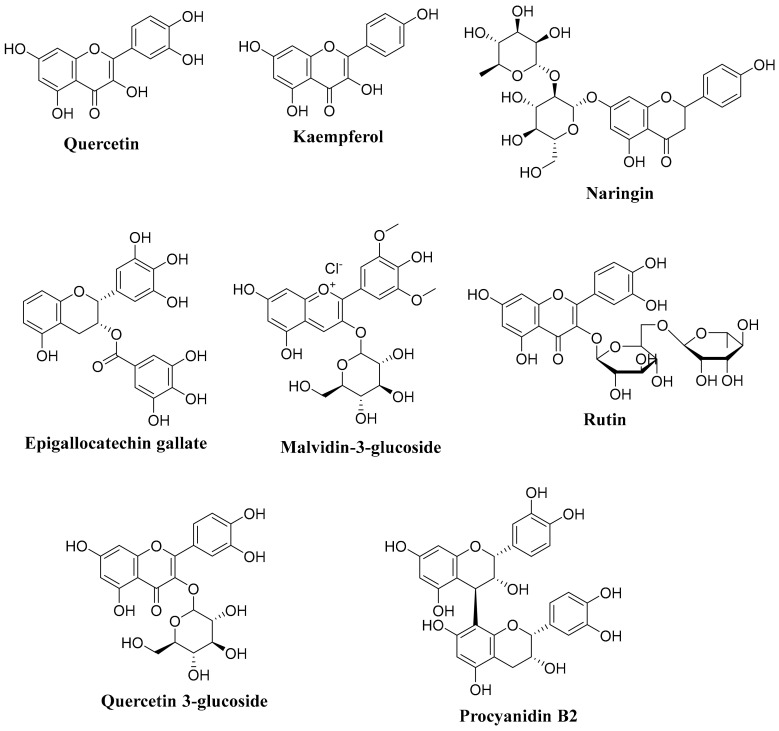

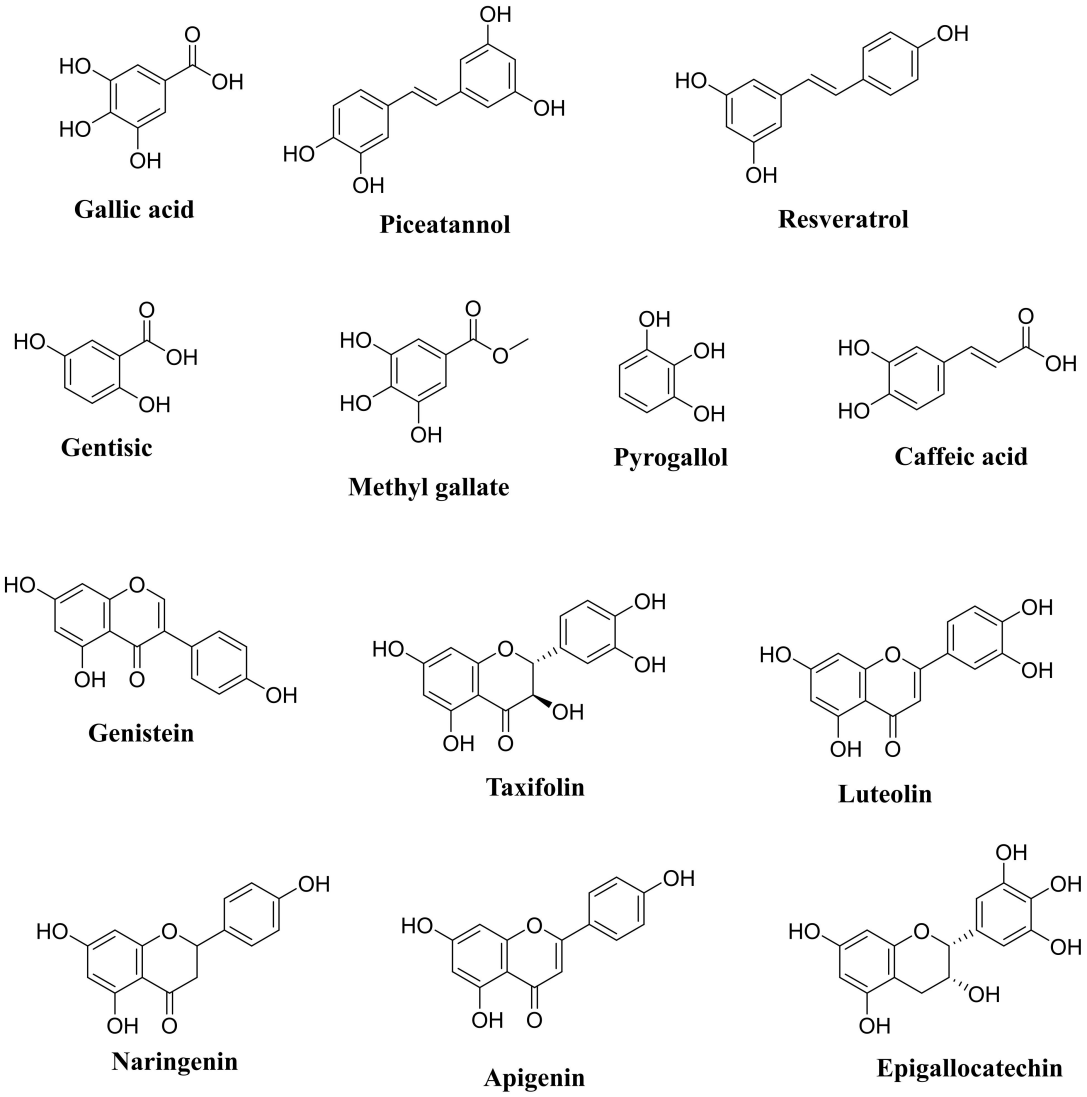
Structures of some important dietary polyphenols.

Dwarf pomegranate peel, juice, and seed-oil extract were investigated for their antiproliferative and apoptotic effects against the human prostate androgen-independent cell line DU145. The cell viability and morphological alterations were detected by MTT assay and light microscopy. At the same time, ELISA and western blotting were used to estimate DNA fragmentation and apoptotic-associated expression of cyclooxygenase-2 (COX-2) and poly-ADP-ribose polymerase (PARP). The half-inhibitory concentration (IC_50_) values of seed oil, juice, and peel extract to exhibit cytotoxic, antiproliferative (by 50%), and morphological alterations in the DU145 cell line were 0.12, 0.36, and 0.42 mg/mL, respectively. The extracts might also enhance PARP cleavage and DNA fragmentation and inhibit COX-2 expression. The peel extract displayed the most substantial pro-apoptotic effect [58]. Listed in Table 2 are active dietary polyphenols, from various fruit and vegetable extracts, against prostate cancer cell lines. Structures of some active dietary polyphenols are shown in Figure 2.

The effect of phenolics from specialty potato cultivars (CO112F2-2, PATX99P32-2, ATTX98462-3, and ATTX98491-3), along with organic, phenolic acid, and anthocyanin fraction (AF), was investigated on androgen-dependent and independent (LNCaP and PC-3) prostate cancer cells. Extracts and AF from the CO112F2-2 cultivar proved to be the most effective in inhibiting cell proliferation and enhancing the cyclin-dependent kinase inhibition-p27, in both LNCaP and PC-3 cells, at 5 μg chlorogenic acid eq mL^−1^. The induction of apoptosis in both cell types was cell context-dependent. The cell death pathways were most likely associated with c-jun N-terminal kinase and mitogen-activated protein kinase stimulation, which ultimately induced caspase-independent apoptosis, through nuclear translocation of endonuclease G (Endo G) and apoptosis-inducing factor [59].

A recombined standard mixture (RSM) of intrinsically present phenolics from *Vaccinium myrtillus* berry extract (VME) was tested against hormone-dependent (LNCaP) and hormone-independent (PC3 and DU-145) prostate cancer (PCa) cell lines, in order to assess its action on growth inhibition and pro-apoptotic activity. Normal prostate epithelial cells (PrEC) were similarly examined for comparison. The dose-dependent VME application caused a decrease in anchorage-dependent PCa cell proliferation with growth inhibition of PrEC. The apoptosis and its rate (non-apoptotic, early, and late apoptosis, as well as necrotic cells, etc.) were significantly higher in the VME-treated cell line than in the control. The growth inhibition patterns of RSM in anchorage-dependent and -independent cells were almost similar to VME [60].

The cancer recurrence after primary treatment depends mainly on the re-entry of quiescent cancer cells into the cell cycle. In this respect, citrus peel extracts (CPE_S_) exhibited significant antitumor effects, though their mechanism of action in regulating the cell cycle is yet unclear. An in vitro culture system was treated with citrus peel ethyl acetate, i.e., hexane and water extracts (CPE-hexane and CPE-water), to regulate the re-entry of quiescent (PC-3 and LNCaP) prostate cancer cells. CPE-water treatment impaired PC-3 and LNCaP cancer cells, causing them to enter the S-phase with 2–3% G0/G1 cells reduction, which was 12–18% in the controls. Contrary to this, CPE-hexane extract was unable to inhibit the cell cycle of both cell lines. CPE_S_ treatment resulted in a low DNA synthesis rate with fragile apoptotic activity in quiescent cancer cells. The predominant flavonoids (hesperidin and narirutin) were not responsible for the observed biological activity. Instead, citric acid was identified as a notable alternative bioactive compound to inhibit re-entry. In PC-3 prostate cancer cells, citric acid exhibited higher cell toxicity than the non-cancerous RWPE-1 prostate cells, signifying its definite benefits in cancer treatment [61].

The nonpolar leaf extracts of the most widely grown Turkish fig cultivars (Sari Lop and Aydin Black) in Aydin were examined for anticarcinogenic potential. In the PC3 human prostate cancer cell line, the *n*-hexane extracts (manual and semi-automated Soxhlet-extracted) caused a cytotoxic effect in a dose-dependent manner, with 100% death at 1000 μg/mL concentration, when applied for 24 h. The extraction method and cultivars have no significant differences in cytotoxic effects [62]. The antioxidant activity, polyphenol, and vitamin C content of the young shoots and mature red cabbage, as well as their comparative in vitro effect on reducing proliferation in prostate cancer cell lines DU145 and LNCaP, were determined. The results revealed that the antioxidant activity, carotenoids, and vitamin C were significantly higher in edible young shoots than in the mature stage with similar polyphenols content. In addition, flavonoids were the significant polyphenols in young shoots, while phenolic acids were predominant in ripe vegetables. Moreover, the juice extracted from young shoots exhibited a more significant antiproliferative effect against cancerous cells than ripe vegetables [63].

The phenolic-rich extracts from feijoa flesh, peel, and whole fruit were tested for their anticancer potential using a human prostate cancer cell line (LNCaP). The extracts exerted a cancer-specific anti-proliferative response by stimulating caspase-dependent apoptosis, sub-G1 phase in the cell cycle, and caspase 3-,8-, and 9 activities, as well as decreasing mitochondrial membrane potential in treated cell lines. The anti-cancer activity of the extracts is most likely attributed to their higher phenolic contents (0.14–0.37 mg GAE/mg DW), especially the ellagic acid (2.662–9.119 μg/mg DW). The activation of caspase-dependent apoptosis suggests phenolic-rich feijoa extracts as a possible nutraceutical food ingredient [64].

An evergreen shrub (*Ixora coccinea*), which bears fruits, is mainly used in the Indian traditional medicine system. A ready-to-serve beverage was developed from these fruits to investigate the phytoconstituents in vitro antioxidant, anticancer, and biological activities. The phenolics, anthocyanin, and flavonoid concentrations, revealed after the phytoconstituents screening, were 128, 30, and 20.63 mg/100 g, respectively. In addition, HPLC analysis identified sinapic acid (21.96 mg) and myricetin (0.13 mg) as the predominant phenolics. The IC_50_ of the fruit extract against the LNCaP.FGC cells effect was 34.09 mg/mL [65]. Published data indicated that the cocoa bean husk (CBH), a significant by-product of the cocoa industry, contains procyanidin-B, catechin, and epicatechin in high concentrations. The anticancer and antioxidant potential of these polyphenols against prostate cancer cells was assessed. In addition, the total polyphenol and flavonoid content, ABTS, DPPH, and radical scavenging potentials of the crude extract in butanol (BF) and ethyl acetate fractions (EAF) were evaluated. HPLC analysis revealed that the procyanidin-B, catechin, and epicatechin concentrations were 20.29, 5.64, and 20.47 mg/g, respectively, and the antioxidants in EAF were comparable to BF. Treatment of PC3 and DU145 cancer cell lines with these fractions, at a dose of 200 μg/mL and annexin V/propidium iodide, caused DNA fragmentation and apoptosis in a dose-dependent manner. The TUNEL assay confirmed the highest phytochemical and antioxidant activity in the EAF fraction [66].

Yellow passion fruit (*Passiflora edulis* sp.) bagasse extract (PFBE) was tested for its antioxidative and anti-inflammatory response in cancer progression using transgenic mice (TRAMP). The extract exhibited a strong in vitro antioxidant capacity and contained scirpusin-B, piceatannol, citric acid, dicaffeoylquinic acid, and (+)-catechin as major bioactive compounds. The notable systemic alterations in the plasma during cancer progression enhanced superoxide dismutase (SOD) and glutathione peroxidase (GP_X_) activities, as well as the malondialdehyde (MDA) and alanine aminotransferase (ALT) levels. Similarly, an increase in MDA, alpha tumor necrosis factor (TNF-α), nuclear transcription factor (NF-κB) levels, glutathione reductase (GR), and GP_X_ activities in the liver were also verified. Furthermore, long- and short-term PFBE administration reduced the MDA levels in the plasma and liver. The plasma catalase activity was increased on long-term PFBE administration, while the short-term administration increased SOD and catalase activities in the liver. Moreover, the long-term treatment reduced the TNF-α and NF-κB levels in the liver [67].

The isolated and methanol-extracted (70%) polysaccharides from *Eriobotrya japonica* fruits were investigated for their cytotoxic potential and chemical composition. The cytotoxic activity was assessed by SRB (Sulforhodamine B) assay, which revealed that the extract exerts moderate activity against the prostate cancer cell line (PC-3), with an IC_50_ of 35 μg/mL. In contrast, the IC_50_ of doxorubicin was 5.48 μg/mL [68]. Similarly, five varieties of jaboticaba were examined for their anthocyanin and non-anthocyanin phenolic compositions, as well as their respective antiproliferative effect on breast and prostate cancers. Cyanidin-3-glycoside and delphinidin-3-glycoside were the most abundant anthocyanins. Additionally, 3 myricetin, 14 quercetin, 13 ellagic acids, and 4 methyl ellagic acid derivatives, with their three forms, were also detected. The Pintada variety, harvested in 2015, exhibited the highest antiproliferative activity [69].

Lycopene, apigenin, and resveratrol from pink guava (*Psidium guajava* L.) were tested for combined and separate effects on LNCaP cells (prostate cancer). Confocal microscopy with anti-PCNA and anti-5-α-reductase antibodies, as well as MTT assays, were used for the first time, in order to establish a ranking for these biomolecules for their separate antiproliferative activity. Apigenin was the most active biomolecule in antiproliferative activity, followed by lycopene and resveratrol. Considering the combined effect of “lycopene-apigenin” and “lycopene-resveratrol,” the “lycopene-apigenin” combination exhibited more potent anticancer activity than the “lycopene-resveratrol” combination [70]. The sweet and hot pepper ethanol extracts were separated into lipophilic fractions. Dried extracts and fractions were subjected to chemical composition, antioxidant potential, and cytotoxic effects against PC-3 and HTC-116 cells. The highest level of phenolic compounds revealed by LC-QTOF-MS analysis was in hot pepper, with the most potent cytotoxic effect on cells. From the 53 identified compounds, only four were present in hot pepper, while six were in sweet pepper. The biological activity of the extracts was accredited to their unique chemical composition, which may be verified experimentally [71].

### 4.2. Colorectal Cancer, Polyphenols, and Identification Techniques

The altered polyphenols in lettuce grown in a reduced nitrogen supply environment were tested for potential anti-proliferative effects against Caco-2 colorectal cancer cells. These polyphenols have shown better anti-proliferative response, interfered with the cell cycle, and induced apoptosis, compared to those grown under ample nitrogen supply. Correlation and HPLC analysis attributed the anti-proliferative response to enhanced percent contribution of quercetin to total polyphenolic [72]. Similarly, the Oriental sweetgum (*Liquidambar orientalis* Mill. var. orientalis (Hammamelidaceae)), a medicinal Turkish endemic plant, was investigated for its molecular and cellular mechanisms and active therapeutic components, in order to treat colorectal cancer. The methanol leaf extract exhibited the highest cytotoxicity in the HCT-116 and HT-29 cell lines, with IC_50_ values of 27.80 and 43.13 μg/mL, respectively, compared to all other extracts. The major phytochemicals identified were quercetin 3-glucoside, pyrogallol, chlorogenic acid, apigenin 7-*O*-glucoside, epigallocatechin gallate, genistein, gallic acid, kaempferol, and luteolin [73].

On the other hand, the methanol extract and isolated compounds from the Porodaedalea pini mushroom were tested for anticancer activity against DLD-1 and CCD-18Co (human colorectal cancer and fibroblast cell line), using the Alamar Blue assay. The results showed that the cytotoxic effect against DLD-1 follows the order: P. pini methanol extract > 4-(3,4-dihydroxyphenyl) but-3-en-2-one (3) > pinoresinol (2) > ergosta-7,24 (28)-dien-3β-ol (1). The methanol extract exerted the best cytotoxic effect against DLD-1 and CCD-18Co cells, with IC_50_ values of 25.33 ± 0.29 and 434.30 ± 1.45 µg/mL, respectively. These findings suggest that the P. pini mushroom could be used as a novel natural therapeutic agent to treat colorectal cancer [74]. Listed in Table 3 are the active polyphenols from various fruit and vegetable extracts against colorectal cancer cell lines.

Similarly, the olive leaf extract was explored for its anti-cancer properties using in vitro HT29 and PC3 colorectal and prostate cancer cell lines. HPLC analysis indicated a significant chlorogenic acid content in the leaf extract. This acid inhibits cancer cell growth, DNA fragmentation, migration, S-phase cell cycle arrest, ROS production, and altered gene expression. Additionally, the extract has a more profound effect on HT29 cells, suggesting it is a promising novel anticancer agent [75].

Similarly, the *n*-Hexane, dichloromethane (DCM), ethyl acetate, *n*-butanol, and water fractions from *Calystegia soldanella* crude extracts were tested for biological activity against HT 29 colorectal cell line. The cell viability assay revealed marked dose- and time-dependent effects of the DCM fraction by enhancing apoptosis and its rate, as well as inhibiting HT 29 cell viability. Furthermore, the extract caused an increase in the apoptosis-related proteins expression levels of Fas, Bad, and Bax, and a decrease in Bcl 2, Bcl xL, procaspase 9, procaspase 8, Bcl 2, procaspase 3, and procaspase 7. Additionally, more cells were depolarized with increased cytochrome c release, S-phase arrest, and reduced cell cycle-related proteins (cyclin A, CDK2, cell division cycle 25 A, and cyclin-dependent kinase inhibitor-1). These results suggest that the DCM fraction of the *C. soldanella* extract can inhibit cancer in HT 29 cells [76]. 

Among the widespread plant species, L, used as a vegetable by folk people, was investigated for its anti-cancer properties against colorectal cancer cells. Six extracts were analyzed for phenolic and flavonoid contents, antioxidant, and cytotoxic activity. The expression difference in the miR-200 family and target genes was determined by real-time qPCR in cells. The anti-proliferative activity was measured by TUNEL, while cell cycle, Annexin V, invasion analysis, and BCL-2, ZEB1, GATA4, and FAS/CD95 protein levels were determined by ELISA. The methanol root extract was the only one that significantly increased the miRNAs (miR-200a/b/c and miR-141) expression in both cell lines and decreased BCL-2, ZEB1, and GATA4 expressions [77].

Olive leaf, destoned fruit, pomace, branch, seed, shell, and extra virgin olive oil from three cultivars, *Frantoio*, *Leccino*, and *Moraiolo*, were investigated for phenolic and triterpenoid contents by HPLC-DAD-MS. Out of 43 identified molecules, oleuropein was a significant constituent in branches, fruits, leaves, and shells with concentrations of 82.72, 55.79, 36.71, and 1.26 g/kg, respectively. Verbascoside (4.88 g/kg and nuzhenide 11-methyl oleoside (90.91 g/kg) was similarly abundant in pomace and seeds. As for triterpenoids, oleanolic acid was present in leaves in the highest amount (11.88 g/kg). Furthermore, the dry extract from the Frantoio cultivar, with the highest phenols and triterpenoids, was selected for a cytotoxicity study against HCT-116 colorectal cells. The results showed that the dry branch extract was the most potent, with an IC_50_ of 88.25 μg/mL, followed by ursolic acid (IC50 24 μM) in a dose-dependent manner [78].

On the other hand, the wild banana (*Ensete superbum* Roxb. Cheesman), traditionally used to treat diarrhea and fever, was preliminarily screened for cytotoxic potential (cell viability of 49% against HCT-15 at 75 µg/mL, as well as 46% against Caco2 at 50 µg/mL) of its ripe-peel (PA), seed (SA), flower (FA), and bract (BA) aqueous extracts. A bioassay-guided technique, with multiple fractions, yielded eight compounds in PA and identified as quercetin-3-*O*-rutinoside, 4′,5,7-trihydroxyflavone, and 3,5-dimethoxy-4-hydroxybenzoic acid by UPLC-HRMS/MS, FTIR, and NMR. The respective cytotoxic activity of these compounds at 10 µg/mL in cell viability against HCT-15 were 50.1, 61.9, and 46.5%. The compounds were also apoptotic as measured by dual staining, mitochondrial membrane potential, ROS status, and DNA fragmentation in colorectal cells [79].

## 5. Conclusions

A significant portion of the world’s population depends on intrinsic foodstuffs in the fight against diseases, such as cancer insurgence. This dependence is gaining popularity, due to the lesser side effects and cost of natural products, compared to synthetic drugs. Therefore, the consumption of phytochemicals, such as polyphenols and their related derivatives, positively correlates with treating cancers. Polyphenols, found in plant foods, are considered some of the most abundant antioxidants in the human diet and play a significant role in the fight against diseases (including cancer). In this review, we have discussed the extraction methods of polyphenols and their bioavailability. In addition, we have shown, through documented published research work, that polyphenols provide a wide range of preventive and therapeutic options against colorectal and prostate cancers, along with a description of the mechanisms by which these compounds exert their action. In summary, this review shows that polyphenols can be a significant complementary medicine for preventing and treating colorectal and prostate cancers. However, more work, including clinical trials on humans of these natural compounds, is needed to establish their efficacy and safety, as well as to search for the best extraction technique to enhance their bioavailability. In addition, we have shown, through documented published research work, that polyphenols provide a wide range of preventive and therapeutic options against colorectal and prostate cancers, along with a description of the mechanisms by which these compounds exert their action. The current challenge in polyphenol research is preserving bioactivity for maintaining food functionality and an optimal delivery system. So, the future represents evolving effective methods for possible polyphenol use, including nanotechnology, chemical modification, encapsulation, lyophilization, emulsions, polyphenol-protein interactions, cold plasma treatment, etc. In summary, this review shows that polyphenols can be a significant complementary medicine for preventing and treating colorectal and prostate cancers, if optimally used to formulate functional foods with enhanced polyphenol stability and delivery. However, more work, including clinical trials on humans, regarding these natural compounds, is needed to establish their efficacy and safety and search for the best extraction technique to enhance their bioavailability.

## Figures and Tables

**Table 1:** Comparison of traditional and modern extraction methods.

Method		Time	Solvent Usage	Cost	Instrument Cost	Sample Size	Efficiency	Polyphenols’ Yield
Traditional	Soxhlet extraction	Moderate	Moderate	Moderate	High	Large	Low	Low
	Maceration	Long	Large	Moderate	Low	Large	Low	Moderate
	Percolation	Moderate	Moderate	Moderate	Low	Large	Moderate	Moderate
Modern	UAE	Short	Little	Low	Low	<30 g	High	High
	MAE	Short	Little	Moderate	High	<10 g	High	High
	SFE	Short	Little	High	High	<5 g	High	High

UAE, ultrasound-assisted extraction; MAE, microwave-assisted extraction; SFE, supercritical fluid extraction.

**Table 2:** Dietary polyphenols, from various fruits and vegetable extracts, against prostate cancer cell lines.

Name of Fruit/Vegetable	Origin	Plant Part	Extraction Solvent	Active Classes/Compounds & Identification Method	Model	Findings	References
Sweet potato (*Ipomoea batatas*)	-	Leaf	Methanol	Anthocyanins	In vitro & in vivo	Active against all tested prostate cancer cell lines, with IC _50_ values in the range of 145–315 μg/mL, reduced the growth and progression of prostate tumor xenografts by ∼69% in nude mice at 400 mg/kg.	[54]
Pomegranate (*Punica granatum*)	United States	Fruit/juice	-	Punicalagin, ellagic acid, gallotannin	In vitro	Caused significant dose-dependent inhibition against the androgen-independent (LNCaP–AR) human prostate cancer cell line.	[55]
Pomegranate (*Punica granatum*)	China	Peel	60% ethanol	Punicalagin, ellagic acid, gallic acid	in vivo	High, medium, and low dosages of pomegranate peel inhibited tumor growth by 41.66, 36.57, and 31.89% percent, respectively, in tumor-bearing mice.	[56]
Pomegranate (*Punica granatum*)	Brazil	Juice/peel	Aqueous	α-punicalagin, β-punicalagin, ellagic acid	In vitro	Pomegranate juice and peel extracts decreased prostate cancer cell growth, migration, and colony formation.	[57]
Pomegranate (*Punica granatum*)	Tunisia	Fruits Peel Seed oil	Methanol	-	In vitro	The IC_50_ dosages for seeds oil, juice, and peel extracts against human prostate cancer cells (DU145) were 0.12, 0.36, and 0.42 mg/mL, respectively, and mediated by a pro-apoptotic mechanism.	[58]
Potato **(***Solanum tuberosum***)**	United States	Tubers	85% ethanol	Anthocyanins		In both LNCaP and PC-3 cells, potato (CO_112_F_2_-_2_ cultivar) extracts, and anthocyanin fraction at 5 g chlorogenic acid eq/mL were active, suppressed cell growth, and increased cyclin-dependent kinase inhibitor (p27) levels.	[59]
Bilberry (*Vaccinium myrtillus*)	Italy	Fruit	80% methanol	Peonidin-3-glucoside, malvidin-3-galactoside, malvidin-3-glucoside, gallic acid, p-coumaric acid, chlorogenic acid, caffeic acid, catechin, epicatechin, phloridizin, myricetin, quercetin,	In vitro	Inhibited the proliferation of both hormone-dependent prostate cancer cells (LNCaP) and hormone-independent (PC3) cells in a concentration-dependent mode. Importantly, normal prostate epithelial cells (PrEC) were resistant.	[60]
Sweet orange (*Citrus sinensis*)	Australia	Peel	Water	Citric acid	In vitro	In the presence of citrus peel water extract (CPEs), quiescent PC-3 and LNCaP cancer cells could not reach the S phase. Quiescent cancer cells treated with CPEs showed reduced DNA synthesis and apoptotic rates.	[61]
Fig (*Ficus carica*)	Leaf	Turkey	n-hexane	-	In vitro	The *n*-hexane extracts exhibited dose-dependent cytotoxic effects on PC3 cells and induced almost 100% death at 1000 μg/mL.	[62]
Red cabbage (*Brassica oleracea* var. *capitata* f. *rubra)*	Shoot juice	Poland	70% methanol	Gallic acid, 4-hydroxybenzoic acid, syringic acid, Chlorogenic acid, caffeic acid, p-coumaric acid, ferulic acid, sinapic acid, catechin, epicatechin, naringin, rutin, kaempferol, myricetin	In vitro	Red cabbage juice more efficiently inhibited the proliferation of prostate cancer cell lines DU145 and LNCaP than shoot extract.	[63]
Feijoa (*Acca sellowiana*)	Fruits	New Zealand	Aqueous ethanol	Gallic acid, catechin, ellagic acid, quercetin	In vitro	The extracts demonstrated anti-proliferative action against the LNCaP cell line by inducing caspase-dependent death.	[64]
Jungle geranium *(Ixora coccinea)*	Fruits	India	Methanol	Sinapic acid, myricetin	In vitro	Fruit extract exhibited an anticancer effect against LNCaP. FGC cells, with an IC_50_ of 34.09 mg/mL.	[65]
Cocoa beans (*Theobroma cacao*)	Husk	Korea	Ethyl acetate and butanol fraction	Catechin, epicatechin, procyanidin B	In vitro	Both fractions triggered apoptosis and DNA fragmentation in PC3 and DU145 cells at 200 g/mL.	[66]
Yellow passion fruit (*P. edulis Sims*)	Residues	Brazil	75% ethanol	Piceatannol, scirpusin-B, dicaffeoylquinic acid, citric acid, catechin	In vitro	Notable alterations in systemic parameters were verified during prostate cancer progression.	[67]
Loquat (*Eriobotrya japonica*)	Fruit	Egypt	70% methanol	Gallic acid, chlorogenic acid, caffeic acid, ellagic acid, ferulic acid, syringic acid catechin, vanillin, naringenin	In vitro	Fruit extract exerted notable inhibition against the prostate cancer cell line (PC-3) with an IC_50_ of 35 μg/mL.	[68]
Jaboticaba (*Plinia cauliflora*)	Fruit	Brazil	Ethanol	Cyanidin-3-glycoside, delphinidin-3-glycoside, quercetin, myricetin ellagic acid	In vitro	Extracts exhibited a significant decrease in cellular proliferation against DU-145 tumor cells.	[69]
Pink guava (*Psidium guajava*)	Fruit	France	-	Apigenin, lycopene, resveratrol	In vitro	The combination of lycopene-apigenin exerted more potent anticancer activity against LNCaP cells than that of lycopene-resveratrol and the separate effect of the biomolecules.	[70]
Sweet & hot pepper (*Capsicum annuum*)	Fruit	-	Ethanol	Gallic acid, gentisic, capsiate, methyl cinnamate, capsidiol luteolin, capsaicin, dihydrocapsaicin, capsianoside I methyl	In vitro	Sweet pepper aqueous fraction displayed higher anticancer activity (IC_50_ of 51 mg/mL) than 40% methanol fraction of hot pepper (IC_50_ of 56 mg/mL) against the prostate tumor cell line (PC-3).	[71]

**Table 3:** Dietary polyphenols, from various fruit and vegetable extracts, against the colorectal cancer cell line.

Name of Fruit/Vegetable	Origin	Plant Part	Extraction Solvent	Active classes/Compounds & Identification Method	Model	Findings	Reference
Red pigmented lettuce *(Lactuca sativa*)	China	Seeds	Ethanol	Caftaric acid, chlorogenic acid, caffeic acid, coumaroylquinic acid, chicoric acid, dicaffeoylquinic acid luteolin, quercetin; HPLC	In vitro	Phenolic extracts from lettuce grown under low nitrogen conditions (LP) exhibited better anti-proliferative effects against Caco-2 cells by interfering with the cell cycle and inducing apoptosis, compared with those from the lettuce supplied with adequate nitrogen.	[72]
Oriental sweet gum *(Liquidambar orientalis Mill. var. orientalis)*	Turkey	Leaves	Methanol	Quercetin 3-glucoside, chlorogenic acid, pyrogallol, epigallocatechin gallate, apigenin 7-*O*-glucoside, gallic acid, genistin, luteolin, kaempferol	In vitro	The leaf methanol extract (LM) of *L. orientalis* showed the highest cytotoxic activity in the HCT-116 (IC_50_ 27.80 μg/mL) and HT-29 (IC_50_ 43.13 μg/mL) cell lines.	[73]
Porodaedalea pini *(Phellinus pini)*	Turkey	Aerial parts	Methanol	Ergosta-7,24(28)-dien-3β-ol, pinoresinol,4-(3,4-dihydroxyphenyl)but-3-en2-one; FT-IR, 1D-NMR, 2D-NMR spectroscopy techniques	In vitro	*P. pini* methanol extract exerted the best cytotoxic activity, with the lowest IC_50_ value on DLD-1 (IC_50_: 25.33 ± 0.29 µg/mL), as compared to isolated compounds.	[74]
Olive *(Olea europaea)*	Saudi Arabia	Leaves	Water	Gallic acid, chlorogenic acid, catechin, methyl gallate, caffeic acid, syringic acid, pyro catechol, rutin, ellagic acid, coumaric acid, vanillin, ferulic acid, naringenin, taxifolin, cinnamic acid, kaempferol; HPLC	In vitro	Inhibited the proliferation of colorectal (HT29) and prostate cancer (PC3), migration, DNA fragmentation, cell cycle arrest at the S phase, production of reactive oxygen species (ROS), and altered gene expression.	[75]
Sea Bindweed *(Calystegia soldanella)*	Korea		Ethanol (dichloromethane fraction)	Hydroxybenzoic acid, hydrosinapinic acid, coumaric acid, quercetin	In vitro	repressed HT-29 cell viability, while inducing apoptosis through mitochondrial membrane potential regulation and S-phase arrest.	[76]
*Syrian rhubarb* *(Rheum ribes)*	Turkey	Roots	Methanol	pyrogallol gallic acid, salicylic acid, 3,4-dihydroxybenzoic acid, epigallocatechin, pyrocatechol, epigallocatehin, taxifolin, 4-*O*-methyl-gallate, ferulic acid, epigallocatechin gallate, daidzein, baicalein, 2-hydroxyxanthone, cis-resveratrol, aloin A, luteolin, chrysin, galangin, epicatechin, procyanidin B2, genistin, formononetin, apigenin, genistein, quercetin-3-*O*- glucoside, quercetin, rutin, kaempferol-7-*O*-glucoside, prunetin, emodin, herbacetin	In vitro	Caused a significant increase in the expressions of miR-200a/b/c and miR-141, and suppressed BCL-2, ZEB1, and GATA4 expressions.	[77]
Olive *(Olea europaea)*	Italy	Branch	90% ethanol	Oleuropein, oleuropein diglucoside taxifolin, taxifolin glucoside, comselogoside isobar; HPLC-DAD-MS	In vitro	The most significant inhibition on the cell’s proliferation was induced by the branch dry extract (IC_50_ 88.25μg/mL).	[78]

## Data Availability

Not applicable.
